# Modulating the electronic structure of atomically dispersed Fe–Pt dual-site catalysts for efficient oxygen reduction reactions†

**DOI:** 10.1039/d3sc00250k

**Published:** 2023-02-23

**Authors:** Wei-Shen Song, Mei Wang, Xiao Zhan, Yan-Jie Wang, Dong-Xu Cao, Xian-Meng Song, Zi-Ang Nan, Li Zhang, Feng Ru Fan

**Affiliations:** a State Key Laboratory of Physical Chemistry of Solid Surfaces, College of Chemistry and Chemical Engineering, Innovation Laboratory for Sciences and Technologies of Energy Materials of Fujian Province (IKKEM), Xiamen University Xiamen 361005 China frfan@xmu.edu.cn zhangli81@xmu.edu.cn

## Abstract

Atomically dispersed catalysts, with a high atomic dispersion of active sites, are efficient electrocatalysts. However, their unique catalytic sites make it challenging to improve their catalytic activity further. In this study, an atomically dispersed Fe–Pt dual-site catalyst (FePtNC) has been designed as a high-activity catalyst by modulating the electronic structure between adjacent metal sites. The FePtNC catalyst showed significantly better catalytic activity than the corresponding single-atom catalysts and metal-alloy nanocatalysts, with a half-wave potential of 0.90 V for the oxygen reduction reaction. Moreover, metal–air battery systems fabricated with the FePtNC catalyst showed peak power density values of 90.33 mW cm^−2^ (Al–air) and 191.83 mW cm^−2^ (Zn–air). By combining experiments and theoretical simulations, we demonstrate that the enhanced catalytic activity of the FePtNC catalyst can be attributed to the electronic modulation effect between adjacent metal sites. Thus, this study presents an efficient strategy for the rational design and optimization of atomically dispersed catalysts.

## Abstract 2

This work reports a strategy to design atomically dispersed Fe and Pt dual-site catalysts to improve the catalytic activity by the electronic modulation effect between adjacent metal sites.

## Graphical abstract

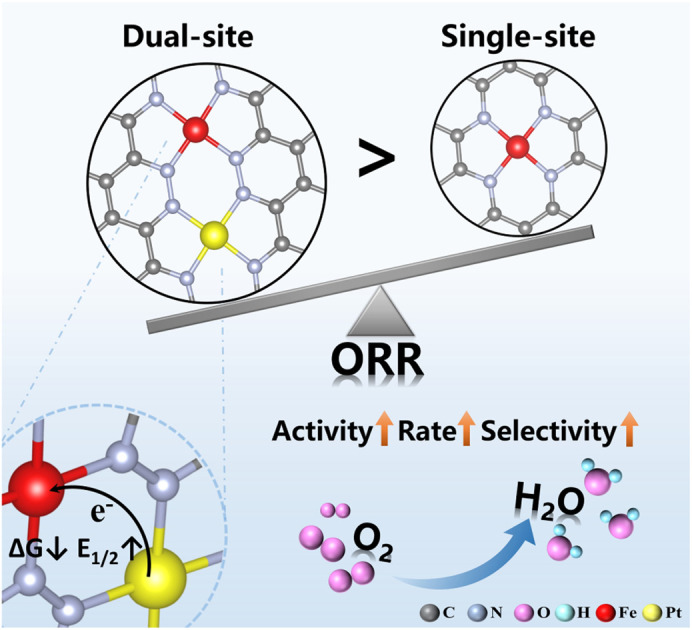

## Introduction

In response to the challenges of global climate deterioration and energy crisis, numerous studies have investigated the development of clean energy conversion devices.^1–3^ Electrochemical energy conversion devices have the advantages of low cost, no pollution, and high energy conversion efficiency. Moreover, electrocatalytic reactions are critical for the collection, storage, conversion, and efficient utilization of renewable energy.^4–7^ At present, Pt-group metal (PGM) catalysts with excellent electrocatalytic performance are always considered the benchmark catalysts for many electrochemical reactions.^8,9^ However, their scarcity, uneven distribution, insufficient stability, and weak anti-poisoning ability limit widespread commercial application.^10^ To overcome this limitation, various other types of electrocatalysts, such as single atom catalysts (SACs),^11–13^ metal alloy catalysts,^14^ and metal core–shell catalysts,^15,16^ have been developed.

In recent years, SACs have gained prominence due to their 100% theoretical atomic utilization efficiency, high selectivity, and excellent activity.^17–21^ Although SACs exhibit outstanding activity in many electrochemical reactions, the simplicity of their active sites and lack of synergistic effects limit the adsorption/desorption Gibbs free energy optimization of intermediate species in catalytic reactions involving multiple electron transfers and intermediate species.^22–24^ The electrochemical oxygen reduction reaction plays a key role in clean and renewable energy conversion and utilization systems, such as polymer electrolyte membrane fuel cells,^7^ metal–air batteries,^25,26^ and H_2_O_2_ electrochemical-production systems.^27^ However, the oxygen reduction reaction (ORR) requires to overcome the high O=O bond energy (498 kJ mol^−1^), which leads to the slow cathode dynamics, severely impeding the industrial application of these techniques.^28^ Moreover, the binding strength of multiple intermediates (OOH*, O*, and OH*) formed by the ORR process to catalytic sites significantly influences the reaction kinetics.^29–31^ FeNC electrocatalysts, consisting of Fe atoms dispersedly embedded in nitrogen-coordinated carbon supports, have been extensively analyzed as PGM alternatives in the ORR.^32^ However, theoretical studies indicate that the electronically symmetrical active centers of FeNC catalysts strongly adsorb OH* (on the Fe sites), adversely affecting the ORR catalytic performance.^33^ In this study, inspired by metal-alloy nanocatalysts, atomically dispersed dual-site catalysts (DACs) were synthesized by introducing a second metal at the adjacent metal active sites of SACs. This strategy maintains the intrinsic activity of single-atom metal sites, while enabling an electronic-state tuning of the catalytic sites by neighboring metal atoms.^34–39^

This study reports a strategy to synthesize ORR catalysts by simultaneously incorporating Fe and Pt single atoms into an N-doped graphene framework (FePtNC) *via* a polymerization-pyrolysis method. Due to the electronic modulation effect between Fe and Pt sites, the fabricated FePtNC catalysts exhibited significantly enhanced ORR performance compared to the corresponding single-atom Fe/Pt catalysts (FeNC, PtNC) and FePt alloy nanocatalysts (FePtNPs). The FePtNC catalysts showed an ORR half-wave potential of 0.90 V (*vs.* RHE), as well as excellent stability and anti-poisoning performance. Batteries containing the synthesized catalyst showed better specific energy density and peak power density (1976 mA h g^−1^ and 90.33 mW cm^−2^, respectively, in Al–air batteries and 713 mA h g^−1^ and 191.83 mW cm^−2^, respectively, in Zn–air batteries) than those containing the commercial Pt/C (20 wt%). Additionally, X-ray absorption spectroscopy and X-ray photoelectron spectroscopy (XPS) were used to confirm the electronic modulation effect of the Fe and Pt atoms (that is, electronic transfer from Pt to Fe) in the FePtNC catalyst. According to density functional theory (DFT) calculations, the Fe sites acted as active centers, while the adjacent Pt sites regulated the Fe-site electronic structure, thereby lowering the energy barrier of the ORR potential-determining step during catalysis. This study provides new insights into the rational design and optimization of atomically dispersed catalysts with high catalytic activity that can overcome the inherent limitations of SACs.

## Results and discussion

### Synthesis and structural characterization of catalysts

**Fig. 1:**
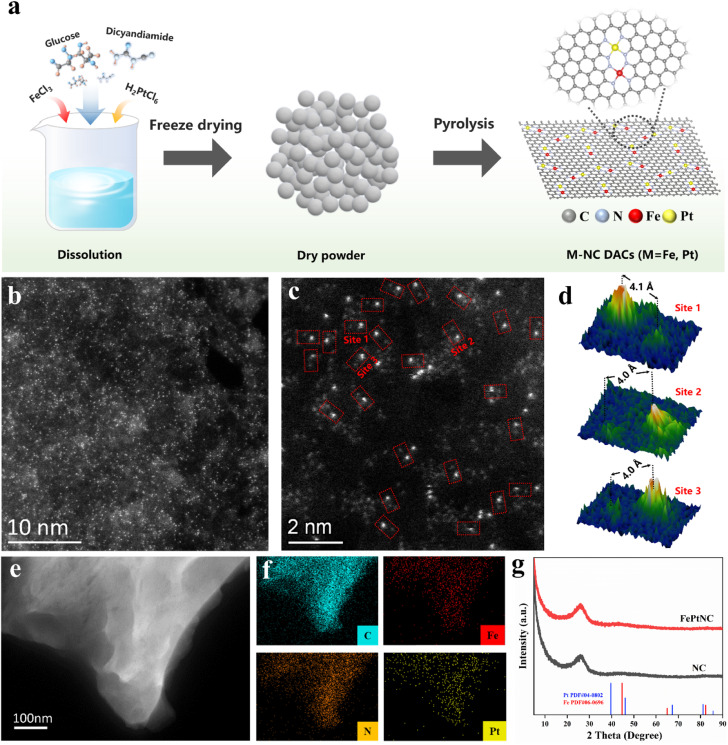
Synthesis and characterization of the FePtNC catalysts. (a) Schematic illustration of FePtNC synthesis. (b and c) Aberration-corrected HAADF-STEM images of FePtNC. The red rectangles in (c) indicate Fe–Pt dual sites. (d) Average intensity profiles of Fe and Pt atoms (indicated by red rectangles) indicating the Fe–Pt distance. (e and f) HAADF-STEM image and corresponding EDS maps of FePtNC. (C, N, Fe and Pt). (g) XRD spectra of FePtNC and NC.

In this study, FePtNC catalysts were fabricated *via* a simple polymerization and pyrolysis process using iron(iii) chloride hexahydrate (FeCl_3_·6H_2_O) and dihydrogen hexachloroplatinate(iv) hydrate (H_2_PtCl_6_·6H_2_O) as the metal precursors, and glucose and dicyandiamide as the C and N sources, respectively (Fig. 1a). Details of the synthesis are provided in the ESI.† Transmission electron microscopy (TEM) and high-angle annular dark-field scanning transmission electron microscopy (HAADF-STEM) images confirmed that FePtNC showed a two-dimensional monolayer or few-layer graphene structure (Fig. 1b and S1†).

Additionally, high-resolution TEM (HR-TEM), HAADF-STEM, and energy-dispersive spectroscopy (EDS) were used to analyze the catalyst microstructure. TEM images of FePtNC confirmed negligible metal nanoparticle or cluster formation on the catalyst surface (Fig. 1b, c and S2b†). Aberration-corrected (AC) TEM was used for atomic-level characterization, to elucidate the atomic structures of Fe and Pt in the catalyst. The AC HAADF-STEM image (Fig. 1b) indicated a large number of bright spots on the carbon support, confirming the uniform distribution of Fe and Pt on the catalyst surface at the atomic level. In the higher-magnification AC HAADF-STEM image (Fig. 1c), a large number of dual-atom metal sites were observed, which have been marked with red rectangles. Based on the *Z*-contrast differences of different metal atoms in HAADF-STEM, the isolated Fe and Pt atoms can be clearly identified by relatively brighter Pt dots and darker Fe dots.^40–42^ Moreover, using different regions of the image, the distances between adjacent Fe–Pt atoms were estimated to be in the range of 4.0–4.1 Å (Fig. 1d). Additionally, the EDS images (Fig. 1e and f) confirmed the uniform distribution of Fe, Pt, N, and C elements on the surface of the FePtNC catalyst, which is consistent with the previous characterization results.

The powder X-ray diffraction (XRD) pattern of FePtNC showed a broad diffraction peak (Fig. 1g), consistent with the peak of graphitic carbon, with no metallic signal. Furthermore, the FePtNC catalysts exhibited a high Brunauer–Emmett–Teller (BET) surface area of 405 m^2^ g^−1^ (Fig. S2†), as indicated by nitrogen adsorption–desorption curves. A high catalyst specific surface area facilitates metal-atom dispersion and stabilization, enabling the efficient transfer of reactants and charges in catalytic reactions. The Raman spectrum of the catalyst showed two distinct oscillation peaks at 1362 and 1578 cm^−1^, which could be attributed to the D-band and G-band peaks of graphitized carbon (Fig. S3†). Moreover, the FePtNC exhibited a high *I*_D_/*I*_G_ ratio of 1.03, indicating a large number of defect sites in the catalyst. Two-dimensional porous and highly defective N-doped materials are ideal supports for atomically dispersed metal-site catalysts.^43^ The metal contents of the synthesized catalysts were analyzed by inductively coupled plasma optical emission spectroscopy (ICP-OES), and the metal loadings of FePtNC were 2.1 wt% (Fe) and 1.45 wt% (Pt), respectively (Table S1†). Furthermore, XPS data also confirmed the presence of Fe and Pt, and Fe 2p_3/2_ (Fig. S4†) and Pt 4f_7/2_ (Fig. S5b†) signals were observed at 710.5 and 72.5 eV, respectively. Additionally, we characterized the structures of FeNC, PtNC, NC and FePtNPs catalysts, as illustrated in Fig. S6–S13.† The NC support material presented a two-dimensional monolayer or few-layer graphene structure. The Fe and Pt metals in the FeNC and PtNC catalysts are distributed on the support at the atomic level, while the FePt metals in the FePtNPs catalyst are present in the form of alloy nanoparticles, with an approximate size of 2 nm.

The chemical environment and information of the catalyst were further analyzed by XPS. In the high-resolution XPS spectrum of FePtNC, the N 1s spectrum was deconvoluted into four components (Fig. S5a†), namely, pyridinic N (398.4 eV), pyrrolic N (399.3 eV), graphitic N (401.4 eV), and oxidized N (402.6 eV), respectively.^44^ The Fe 2p spectra (Fig. S4†) of both FePtNC and FeNC indicated that Fe existed in a non-zero oxidation state. Moreover, no response signal of the zero valence state was observed, confirming the existence of an N-coordinated Fe. Additionally, the peak of Fe in FePtNC was positively shifted compared to that in FeNC, indicating a higher oxidation state of Fe in the former, along with electron transfer from Pt to Fe.^7^ The Pt 4f spectra of FePtNC and PtNC (Fig. S5b and S15†) indicated the presence of Pt with an oxidation state between 0 and +4.

**Fig. 2:**
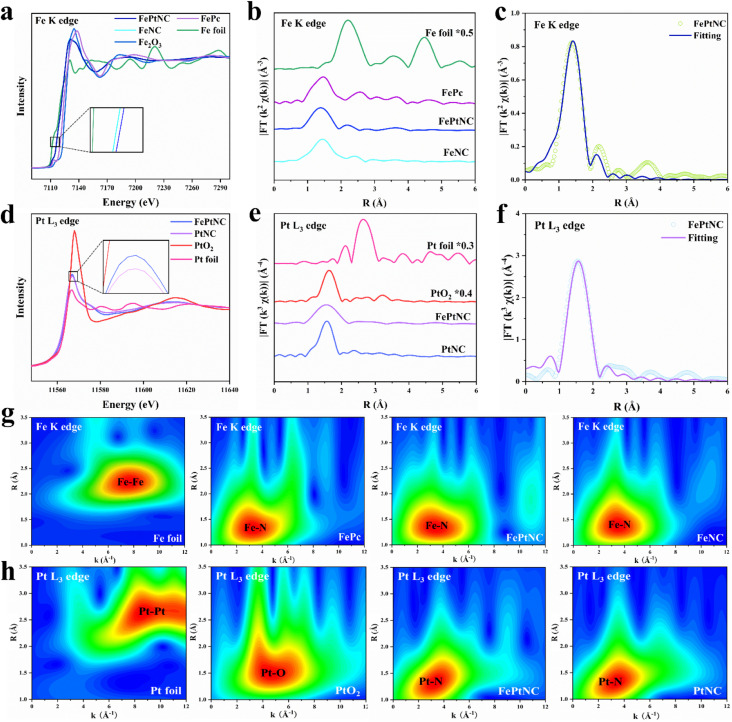
Structural characterization of FePtNC by X-ray absorption spectroscopy. (a) Normalized XANES spectra at the Fe K-edge of FePtNC with those of reference samples. (b) Magnitude of the Fourier transforms of EXAFS spectra of catalysts at the Fe K-edge. (c) Corresponding Fe K edge EXAFS R space fitting curves of FePtNC. (d) Normalized XANES spectra at the Pt L_3_-edge of FePtNC with reference samples. (e) Magnitude of the Fourier transforms of EXAFS spectra of catalysts at the Pt L_3_-edge. (f) Corresponding Pt L_3_ edge EXAFS R space fitting curves of FePtNC. (g and h) Wavelet transform (WT)-EXAFS data of the catalyst and reference sample.

X-ray absorption near edge structure (XANES) and extended X-ray absorption fine structure (EXAFS) spectra were used to analyze the local coordination structures and chemical states of Fe and Pt in FePtNC.^45^ The absorption edge of FePtNC at the Fe K-edge (Fig. 2a) was located between the Fe foil and Fe_2_O_3_, indicating the valence of Fe in FePtNC to be between 0 and +3. Similarly, a comparison of the Pt L_3_ edge spectrum of FePtNC (Fig. 2d) with those of Pt foil and PtO_2_ showed that the line intensity peak was located between the Pt foil and PtO_2_, which indicated that Pt in FePtNC exhibited a positive valence state. As shown in the inset of Fig. 2a, the rising edge of the Fe K-edge spectrum of FePtNC was negatively shifted compared to that of FeNC, indicating a higher oxidation state of Fe atoms in FePtNC compared to that in FeNC, consistent with the XPS results. Additionally, the white line peak for FePtNC at the Pt L_3_ edge showed a stronger intensity than that of the PtNC sample (inset of Fig. 2d), possibly due to electron transfer from Pt to Fe.

Subsequently, an extended edge Fourier Transform (FT) analysis was conducted using Fe K-edge and Pt L_3_ edge spectra of FePtNC and the reference samples. According to FT-EXAFS spectra (Fig. 2b) of the Fe K-edge, the Fe foil and Fe phthalocyanine (FePc) references showed *R* space positions of 2.21 and 1.47 Å, respectively, which could be assigned to Fe–Fe and Fe–N scattering paths, respectively. FePtNC and FeNC showed a single predominant peak at 1.46 Å, which could be assigned to the scattering of the first shell Fe–N path signal. Notably, their spectra contained no peaks at around 2.21 Å, confirming the atomic dispersion of Fe. A detailed analysis of the Pt species in FePtNC is shown in Fig. 2e. The spectra of Pt foil and PtO_2_ showed Pt–Pt and Pt–O signals at 2.65 and 1.63 Å, while a primary peak at 1.56 Å was observed in the *R* space, confirming Pt–N to be the main coordination linkage in FePtNC.

A least-squares EXAFS fit was used to obtain the quantitative chemical configurations of the Fe and Pt atoms. Using the fitting results, the coordination number of Fe and average bond length were estimated to be 4 and 1.99 Å, respectively (Fig. 2c and Table S2†). Additionally, the coordination number of Pt was estimated to be 4, with a mean bond length of 2.06 Å (Fig. 2f and Table S2†). Thus, the local atomic structure of FePtNC consisted of the atomic dispersion of Fe and Pt sites in an M–N_4_ structure. The Fe K-edge and Pt L_3_ edge EXAFS oscillations of FePtNC were analyzed using the wavelet transform (WT) method with high resolution in both the *k* and *R* spaces (to facilitate the distinction between metal, N, and metal–metal bonds).^46^ Several standard Fe (Fe foil and FePc) and Pt (Pt foil and PtO_2_) specimens were used as references. The *k*-space intensity maxima of FePtNC and FeNC were 3.2 Å^−1^, while those of Fe foil and FePc were 7.5 and 3.4 Å^−1^, respectively (Fig. 2g). Similarly, in the analysis of Pt species, the *k*-space intensity maxima of FePtNC and PtNC were 3.4 Å^−1^, and those of Pt foil and PtO_2_ are 8.6 and 4.4 Å^−1^ (Fig. 2h). The results further demonstrate that the metal Fe and Pt sites in the FePtNC catalyst exist in an atomically dispersed form.

### Electrocatalytic oxygen reduction performance

**Fig. 3:**
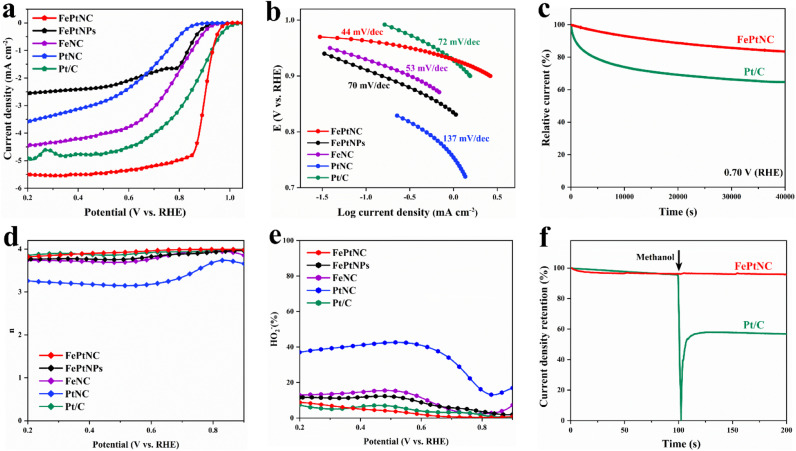
ORR performances of FePtNC in O_2_-saturated KOH (0.1 M). (a) ORR polarization curves of FePtNC, FePtNPs, FeNC, PtNC, and commercial Pt/C catalysts. (b) Corresponding Tafel plots obtained from the ORR polarization curves. (c) *I*–*t* curve for FePtNC and its comparison with that of commercial Pt/C at 0.70 V *vs.* RHE. (d and e) Electron transfer number and HO_2_^−^ yield ORR polarization curves. (f) Methanol tolerance test of FePtNC and commercial Pt/C.

To investigate the ORR catalytic performance of the electrocatalyst, its performance was analyzed in an oxygen-saturated KOH (0.1 M) solution using a rotating disk electrode (RDE). Control experiments with FeNC, PtNC, and FePtNPs were used to explore the advantageous properties of the FePtNC dual-site catalyst. As shown in Fig. 3a, linear sweep voltammetry (LSV) indicated that the FePtNC catalysts showed a better oxygen-reduction performance (with a half-wave potential (*E*_1/2_) of 0.90 V) than commercial Pt/C (*E*_1/2_ = 0.87 V). Moreover, the *E*_1/2_ value of the FePtNC catalyst was higher than those of FeNC, PtNC, and FePtNPs, confirming the higher catalytic activity of DACs compared to those of SACs and metal-alloy catalysts. Tafel slopes from LSV curves were used to evaluate the ORR kinetics of the catalysts. As shown in Fig. 3b, among all the samples analyzed, FePtNC showed the fastest ORR kinetics with the smallest Tafel slope of 44 mV dec^−1^.

Subsequently, the catalyst stability was analyzed by a chronoamperometric test and an accelerated durability test. As shown in Fig. 3c, the FePtNC catalyst exhibited better stability than the commercial Pt/C catalyst after testing in an oxygen-saturated electrolyte for 40 000 s. Furthermore, the FePtNC showed robust stability with only a negative shift of 8 mV on *E*_1/2_ after 10 000 consecutive cycles (Fig. S24†). Examination of XRD, TEM, and HAADF-STEM results (Fig. S25 and S26†) confirmed that the original structure of the catalyst remained after the durability testing. A rotating ring-disk electrode (RRDE) was used to analyze the catalytic pathway of the catalyst during the oxygen reduction process. As indicated by ring and disk currents, the FePtNC catalyst exhibited a low H_2_O_2_ yield and high electron transfer number (Fig. 3d and e), indicating a 4e^−^ transfer pathway to be predominantly operative. Furthermore, the methanol tolerance of FePtNC was evaluated by injecting a methanol solution during chronoamperometry testing. Significant current density retention was not exhibited by FePtNC (Fig. 3f), whereas the current density of commercial Pt/C showed a sharp reduction, indicating that FePtNC showed excellent methanol tolerance. We speculate that the reason for the excellent ORR catalytic performance of FePtNC relative to its counterparts is due to the electron modulation effect between adjacent dual-atom sites, while relative to the FePtNP catalyst it is due to the full exposure of atomic sites and unique coordination structure.

### Theoretical analysis of the Fe–Pt dual-site structures

**Fig. 4:**
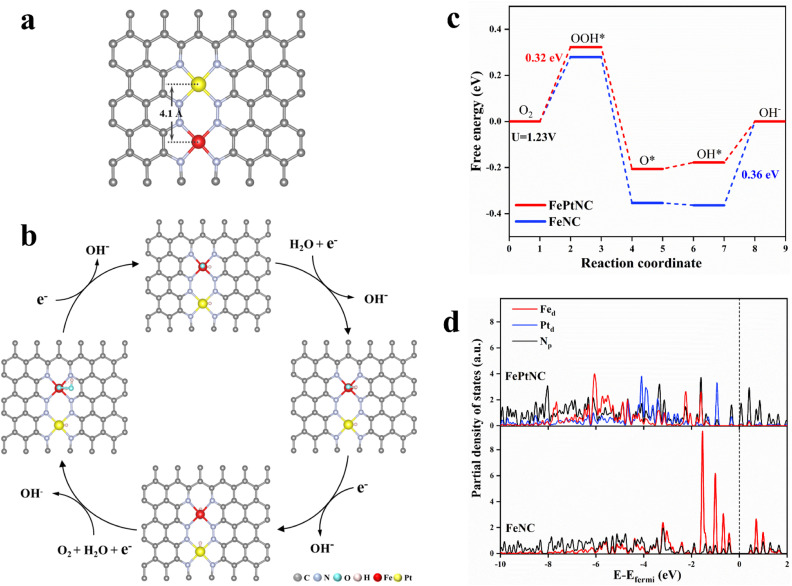
Origin of ORR activity in the Fe–Pt dual-site catalyst. (a) DFT-optimized configuration of FePtNC. (b) Simulated pathways for ORR on the FePtNC catalyst. (c) DFT-calculated free energy diagram of ORR for the FePtNC catalyst. (d) Partial density of states (PDOS) for the d orbitals of Fe/Pt and p orbitals of N.

Density functional theory (DFT) calculations were used to elucidate the properties of the synthesized dual-site catalyst, including the electronic modulation effect between adjacent dual-atom sites. Several possible structural models were proposed for the FePtNC catalyst (Fig. S27†) to identify the optimum model to explore the ORR catalytic mechanism. Subsequently, by combining the geometric configuration of the structural models and experimental results (atomic distance and coordination number), Fig. 4a was selected as the optimal FePtNC dual-site catalyst research model, with ∼4.1 Å distance between adjacent Fe and Pt atoms. Based on this model, a possible reaction pathway for FePtNC was proposed to elucidate the enhanced activity of the dual-site catalyst, as shown in Fig. 4b. First, pure Fe–N_4_–Pt–N_4_ was considered to be the active site. Due to strong interactions between the Fe sites and ORR intermediates, especially OH, the desorption of OH* to OH^−^ was an endothermic reaction, and acted as the potential-determining step (PDS) of the entire reaction. Thus, the calculated overpotential was significantly higher than the experimental value, as shown in Fig. S28.† Therefore, the hydroxyl-attached metal site was identified as the true active site (Fig. S29†). The calculated free energy diagrams (Fig. 4c) indicated weak interactions between the Fe sites and ORR intermediates in the FePtNC catalyst, with the OOH* intermediate-formation step as the PDS (Δ*G*_PDS_ = 0.32 eV). Contrarily, the OH* intermediate-desorption (on the Fe site) step was the PDS (Δ*G*_PDS_ = 0.36 eV) in the FeNC catalyst, indicating a strong binding of the Fe site to OH*. This phenomenon could be attributed to the modulation of the electronic properties of the Fe active sites by adjacent Pt sites in the FePtNC catalyst, thereby optimizing the adsorption/desorption free energy of the intermediate species in the ORR.

Additionally, the partial density of states (PDOS) was used to analyze differences in the electronic properties of the FeNC and FePtNC model surfaces. As shown in Fig. 4d, the Pt-containing catalyst (FePtNC) showed a clear negative energy shift of the electron-cloud state at the Fe site compared to FeNC, possibly due to electron transfer from Pt to Fe through the N bridge in the former. Additionally, this electron transfer weakened the interactions between the Fe active sites and ORR intermediate species, thereby enhancing the catalytic activity. The presence of Pt in the FePtNC catalyst significantly reduced its PDS value during ORR compared to that of the FeNC catalyst by changing the d-electron orbitals of the Fe active sites, significantly accelerating the ORR process.

### Metal–air battery system analysis

**Fig. 5:**
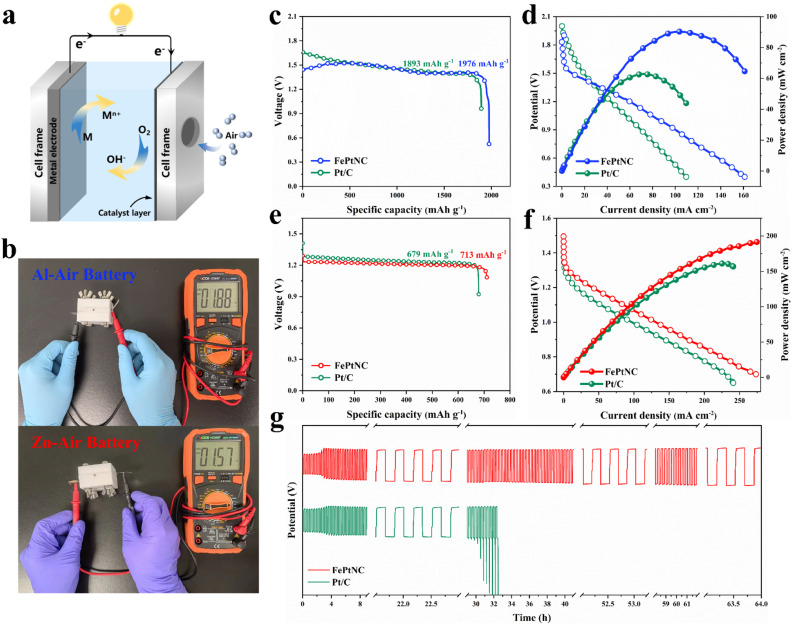
The performance of metal–air battery systems with the FePtNC catalyst. (a) Schematic diagram of the metal–air battery. (b) The open circuit voltage of the metal–air batteries with the PtFeNC catalyst as the air electrode. (c) Discharge profiles of the Al–air battery at 10 mA cm^−2^. (d) Charge/discharge polarization profiles and power density curves of the Al–air battery. (e) Discharge profiles of the Zn–air battery at 10 mA cm^−2^. (f) Charge/discharge polarization profiles and power density curves of the Zn–air battery. (g) Galvanostatic discharge–charge cycle profiles of the Zn–air battery.

As electrocatalytic tests do not accurately represent the performance of a catalyst in practical energy storage devices, a metal–air battery (Fig. 5a) was assembled with an electrocatalyst-loaded carbon cloth as the positive electrode (cathode) and metal foil as the negative electrode (anode), and used for experimentation. The Al–air battery with Al foil as the anode and FePtNC as the air cathode exhibited a high open-circuit potential (OCP) of up to 1.88 V (Fig. 5b). Additionally, the FePtNC-based battery showed a higher specific capacity (1976 mA h g^−1^) than a commercial Pt/C battery (1893 mA h g^−1^) at a current density of 10 mA cm^−2^ (Fig. 5c). Notably, discharge polarization curves and the corresponding power density curves (Fig. 5d) indicated that the FePtNC-based battery showed a higher power density (90.33 mW cm^−2^) than a commercial Pt/C battery (62.85 mW cm^−2^).

Subsequently, Zn–air battery devices (with FePtNC as the air-cathode electrocatalyst and Zn foil as the anode) were used to evaluate the performance of the electrocatalyst in rechargeable batteries. The OCP of FePtNC (1.57 V) (Fig. 5b) was higher than that of commercial Pt/C (1.51 V). Furthermore, the FePtNC catalyst exhibited a higher specific capacity (713 mA h g^−1^) than a commercial Pt/C battery (679 mA h g^−1^) at 10 mA cm^−2^ (Fig. 5e), a significantly higher maximum power density (191.83 mW cm^−2^) than commercial Pt/C (161.20 mW cm^−2^) (Fig. 5f), and a higher open-circuit potential and energy power density than commercial Pt/C. Additionally, the rechargeability and cycling stability of the Zn–air battery were evaluated using galvanostatic charge and discharge measurements. As indicated by a long-term charge–discharge cycling test at 10 mA cm^−2^ (Fig. 5g), FePtNC exhibited excellent cycling stability (64 h) compared to commercial Pt/C (33 h). Moreover, the charge–discharge voltage gap of the former did not increase significantly after the cycling test, indicating that the FePtNC-based Zn–air battery showed excellent cycling stability. Therefore, the metal–air batteries fabricated here show significantly improved performance parameters, such as discharge voltage, specific capacity, and power density, compared to recently reported metal–air batteries using atomically dispersed catalysts as cathodes (Table S4†).

## Conclusions

Summarizing, this study provides a new strategy for optimizing the catalytic performance of atomically dispersed catalysts by introducing adjacent heterometallic sites. A facile and general method was used to precisely synthesize an atomically dispersed Fe–Pt dual-site catalyst. Subsequently, the activity of the synthesized DACs was compared with that of the corresponding single-atom and metal-alloy catalysts. The FePtNC catalyst showed excellent catalytic performance compared to the other catalysts analyzed. Moreover, metal–air battery systems with the catalyst showed peak power density values of 90.33 mW cm^−2^ (Al–air) and 191.83 mW cm^−2^ (Zn–air). According to theoretical simulations, the enhanced catalytic activity of the DACs could be attributed to the electronic modulation effect between adjacent metal sites, which was confirmed by experimental data. Therefore, this study confirms that atomically dispersed dual-site catalysts exhibit high potential for catalytic reactions, and it opens new frontiers in the development of optimized atomically dispersed catalysts.

## Data availability

The data supporting the findings of this study are available within the article and in the ESI.†

## Author contributions

Wei-Shen Song conceived the idea and wrote the manuscript. Wei-Shen Song performed all synthetic experiments and analyzed data. Mei Wang, Xiao Zhan, Yan-Jie Wang, Dong-Xu Cao, Xian-Meng Song, and Zi-Ang Nan analyzed data, performed battery system studies and computational studies, and reviewed the manuscript. Li Zhang, and Feng Ru Fan supervised the project, performed formal analysis, and wrote and reviewed the manuscript. All the authors discussed the results and commented on the manuscript. All authors have given approval to the final version of the manuscript.

## Conflicts of interest

There are no conflicts to declare.

## Supplementary Material

SC-014-D3SC00250K-s001
