# Supplementary material for: Modulating the electronic structure of atomically dispersed Fe–Pt dual-site catalysts for efficient oxygen reduction reactions
Source: Chem Sci. 2023 Feb 23;14(12):3277–85. doi: 10.1039/d3sc00250k (PMC10034214; doi:10.1039/d3sc00250k)
Supplement: SC-014-D3SC00250K-s001 [file SC-014-D3SC00250K-s001.pdf]

Electronic Supplementary Material (ESI) for Chemical Science.
This journal is © The Royal Society of Chemistry 2023

Supplementary Information

Modulating the Electronic Structure of Atomically Dispersed Fe-Pt Dual-Site Catalysts for Efficient Oxygen Reduction Reactions

Wei-Shen Song[a], Mei Wang[a], Xiao Zhan[a], Yan-Jie Wang[a], Dong-Xu Cao[a], Xian-Meng Song[a], Zi-Ang Nan[a], Li Zhang*[,a], and Feng Ru Fan*[,a]

[1]State Key Laboratory of Physical Chemistry of Solid Surfaces, College of Chemistry and Chemical Engineering, Innovation Laboratory for Sciences and Technologies of Energy Materials of Fujian Province (IKKEM), Xiamen University, Xiamen 361005, China.

***e-mail:** zhangli81@xmu.edu.cn; frfan@xmu.edu.cn

1. Experimental Section

Reagents and Chemicals

Iron(III) chloride hexahydrate (99%, Aladdin); Chloroplatinic(IV) acid hexahydrate (AR, Sinopharm Chemical); Glucose (96%, Sigma-Aldrich); Dicyandiamide (99%, Sigma-Aldrich); Potassium hydroxide (99%, Aladdin); Zn foil (99.999%, Alfa Aesar); Al foil (99.9995%, Alfa Aesar); Zinc acetate dihydrate (98%, Sigma-Aldrich); Sodium stannate (95%, Sigma-Aldrich); Methanol (99.7%, Sinopharm Chemical); Ethanol (AR, Sinopharm Chemical); Commercial Pt/C (20 wt%, Johnson Matthey); Nafion (5%, DuPont); Carbon cloth (Cetech); Deionized water (18.2 MΩ, Lab homemade); H_2/Ar (10% H_2, Linde); Ar (99.999%, Linde).

Synthesis of Catalysts

The FePtNC catalysts were synthesized by the following method. Firstly, 0.13 g glucose and 2.5 g dicyandiamide were dissolved in 10 mL deionized water with 80°C water bath heating. After vigorous stirring for 1 hour, the metal salt mixture (5 mg $FeCl_3 \cdot 6H_2O$ and 1 mg $H_2PtCl_6 \cdot 6H_2O$ were dissolved in 2 mL deionized water) was slowly dropped into the transparent solution, and then continue stirring for 12 h. Secondly, the bottle containing the above solution was quickly placed in a liquid nitrogen environment for 0.5 hours, and then freeze-dried. Finally, ground by mortar, the powder was heated at 550 °C for 1 h, then heated at 900 °C for 2 h under argon atmosphere, and cooled to room temperature naturally. The FeNC catalysts were synthesized by the same steps except without the addition of $H_2PtCl_6 \cdot 6H_2O$. The PtNC catalysts were synthesized by the same steps except that $FeCl_3 \cdot 6H_2O$ was not added. The NC catalysts were synthesized with the same steps except that the metal salt was not added.

The FePtNPs catalysts were synthesized by the following method. Firstly, the prepared NC was dissolved in 10 mL of deionized water, and then the metal salt mixture (5 mg $FeCl_3 \cdot 6H_2O$ and 1 mg $H_2PtCl_6 \cdot 6H_2O$ were dissolved in 2 ml of deionized water) was added to the solution. After vigorous stirring for 12 h, the solution was dried at 60 °C. Finally, ground by mortar, the powder was heated at 600 °C for 2 h under hydrogen

atmosphere (10 % H_2, 90 % Ar), and then cooled to room temperature naturally.

Materials Characterizations

The TEM, AC-HAADF STEM and EDS mapping were acquired by using spherical aberration corrected Titan Cubed Themis G2 300 TEM (300 KV) and FEI Tecnai F30 TEM (300 KV). The powder XRD was performed on an X-ray diffractometer (Rigaku, Ultima-IV) with a Cu Kα radiation source (scanning speed, 10°min^{-1}). The XPS spectra were obtained on an X-ray photoelectron spectrometer (Thermo Scientific ESCALAB Xi+); The metal content of catalysts was measured by ICP-OES (SPECTRO SPECTROBLUE FMX36); SEM images were obtained on a field emission scanning electron microscopy (Zeiss GeminiSEM 500); The BET specific surface area is calculated by nitrogen adsorption and desorption isotherms (Micromeritics, TriStar II 3020); The Raman spectra were collected in a laser confocal Raman microscopy system (Nanophoton Corporation), with 532 nm laser as excitation source; XAFS measurements were performed at the XAFS Beamline in the Australian Synchrotron (ANSTO) in Melbourne, Australia. A Ge 100 element detector was used to collect the fluorescence signal, and the energy was calibrated using Fe and Pt foil. The beam size was about 1 mm^2. The XAFS data were processed using Athena and Artemis of Demeter software packages.[1]

Electrochemical Measurements

The electrochemical ORR measurements were performed through an electrochemical workstation (CHI 660E) with a typical three-electrode system. A rotating disk electrode (RDE) loaded with catalyst ink was used as the working electrode, graphite rod as the counter electrode and Ag/AgCl as the reference electrode, respectively. The catalyst ink was prepared by mixing 5 mg catalyst, 50 μL Nafion solution (5%) and 950 μL ethanol with sonication. Then 20 μL catalyst ink was slowly dripped onto the RDE surface and dried naturally. The LSV curves of catalysts were tested in an oxygen-saturated 0.1M KOH solution while keeping the speed of RDE stable at 1600 rpm. The stability of the catalyst was measured in an oxygen-saturated 0.1 M KOH solution at 0.7 V (vs. RHE). All potential values in electrochemical measurements were calibrated

to the reversible hydrogen electrode. (Figure S22)

The electron transfer number and ORR reaction kinetics were calculated based on the Koutecký-Levich equation:

$$\frac{1}{j} = \frac{1}{j_d} + \frac{1}{j_k} = \frac{1}{B\omega^{\frac{1}{2}}} + \frac{1}{j_k}$$

$$B = 0.62nFC_0D^{\frac{2}{3}}\upsilon^{-\frac{1}{6}}$$

In these equations, j_d is the diffusion-limiting current density and j_k is the kinetic current density. The ω is the angular velocity (rad s^{-1}). n is the transferred electron number of ORR pathway. F is the Faraday constant (96485 C mol^{-1}). C_0 is the saturated concentration of O_2 in 0.1 M KOH solution (1.2 * 10^{-6} mol cm^{-3}). D is the diffusion coefficient of O_2 in 0.1 M KOH solution (1.9 * 10^{-5} cm^2 s^{-1}). υ is the kinematic viscosity of the electrolyte (0.01 cm^2 s^{-1}).

According to the following formulas, the yield of H_2O_2 and the transfer number of electrons in the ORR of the catalyst were calculated. Data was obtained from using the rotating ring-disk electrode (RRDE) tested.

$$H_2O_2\,\% = 200 * \frac{\frac{I_r}{N}}{I_d + \frac{I_r}{N}}$$

$$n = 4 * \frac{\frac{I_d}{N}}{I_d + \frac{I_r}{N}}$$

In these equations, I_d is the disk current, I_r is the ring current and N is the current collection efficiency of the Pt ring (N = 0.37).

Computational details and models

All of the computations were performed by Vienna ab-initio simulation package (VASP) with the projector augmented wave pseudo-potentials (PAW) to describe the interaction between atomic cores and valence electrons with density functional theory

(DFT). The Perdew-Burke-Ernzerhof (PBE) functional within the generalized gradient approximation (GGA) were used to implement DFT calculations.[2] In order to simulate the catalyst surface, a 6 × 6 periodic graphene supercell could ensure the reliability of the conclusions in this work. And we confirmed that it exhibits catalytic character in PBE+U theories.[3] The reasonable vacuum layers were set around 15 Å in the z-directions for avoiding interaction between planes. A cutoff energy of 450 eV was provided and a 2×2×1 Monkhorst Pack k-point sampling was chosen for the well converged energy values. Geometry optimizations were pursued until the force on each atom falls below the convergence criterion of 0.02 eV/Å and energies converged within 10^{-5} eV.

Zn-air Battery Measurements

Zn-air battery was assembled using an Zn foil as the negative electrode material, the carbon cloth loaded with the catalyst (1 mg cm^{-2}) as the positive electrode material and 6 M KOH/0.2 M $Zn(OAc)_2$ as the electrolyte, respectively. The preparation process of the catalyst-loaded carbon cloth is that the catalyst is dissolved in 450 μL deionized water, 450 μL ethanol and 100 μL Nafion solution (5%), and then ultrasonically mixed evenly. Finally, the prepared catalyst ink is slowly added to the surface of the carbon cloth and dried naturally. The performance measurements of the battery were carried out in CHI 660E and LAND systems, respectively.

Al-air Battery Measurements

Al-air battery was assembled with a similar process to the Al-air battery, except using Al foil as the negative electrode material, and 4 M NaOH/0.05 mM $Na_2SnO_3 \cdot 3H_2O$ as the electrolyte.

2. Supplementary Figures and Tables

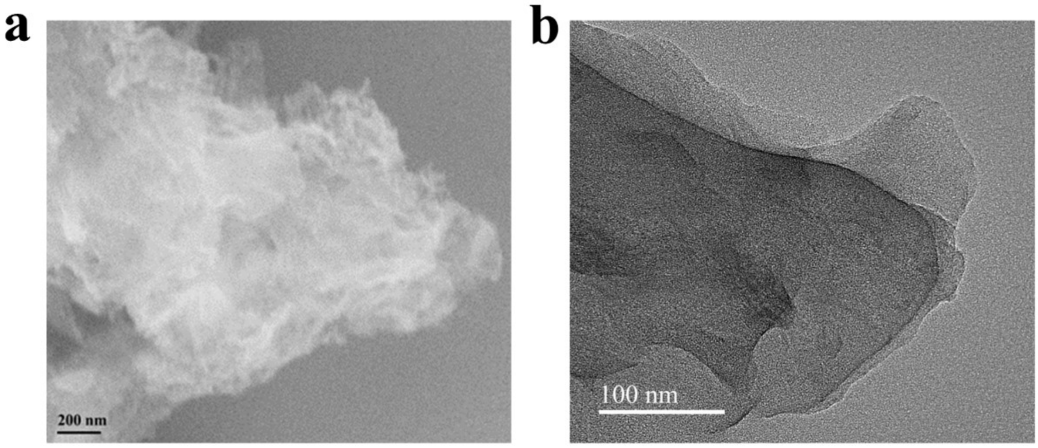

Figure S1. SEM (a) and TEM(b) images of FePtNC.

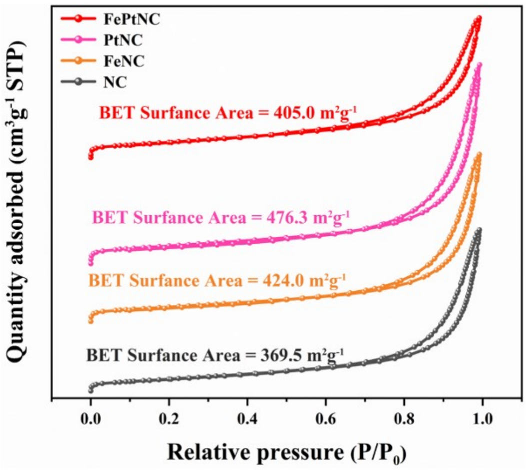

Figure S2. BET specific surface area and nitrogen adsorption-desorption isotherms of catalysts.

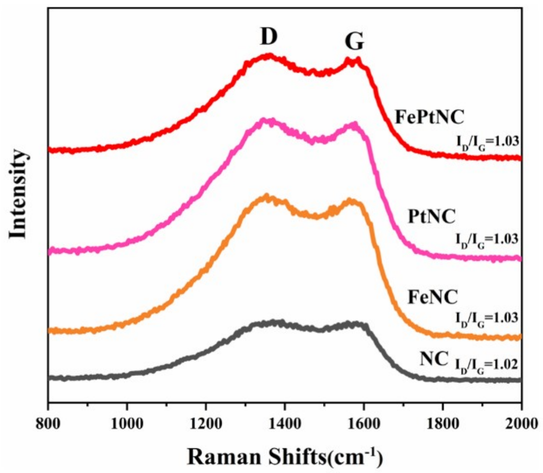

Figure S3. Raman spectra of catalysts.

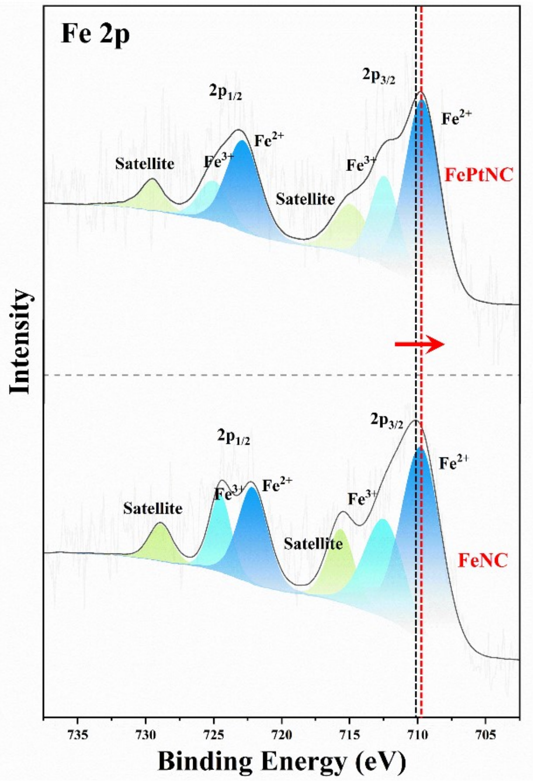

Figure S4. XPS spectrum of FePtNC and FeNC (high-resolution Fe 2p spectrum).

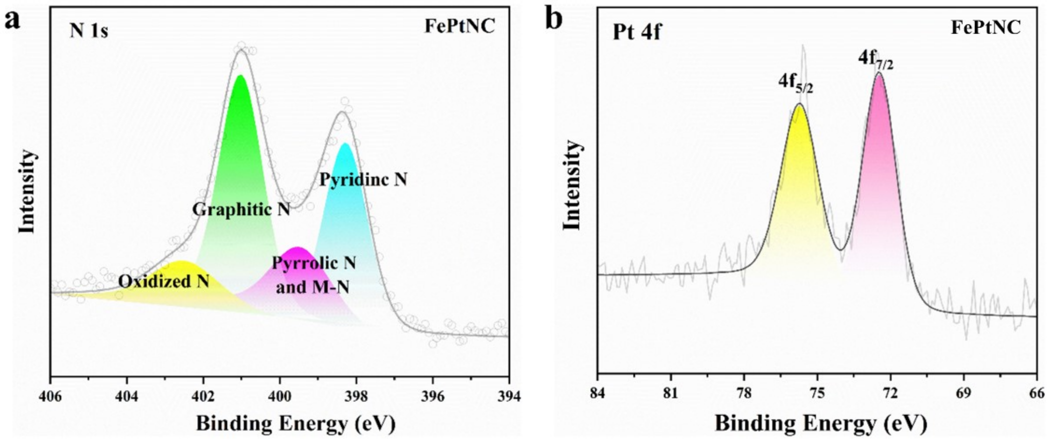

Figure S5. XPS spectra of FePtNC. (a) High-resolution N 1s spectrum; (b) High-resolution Pt 4f spectrum.

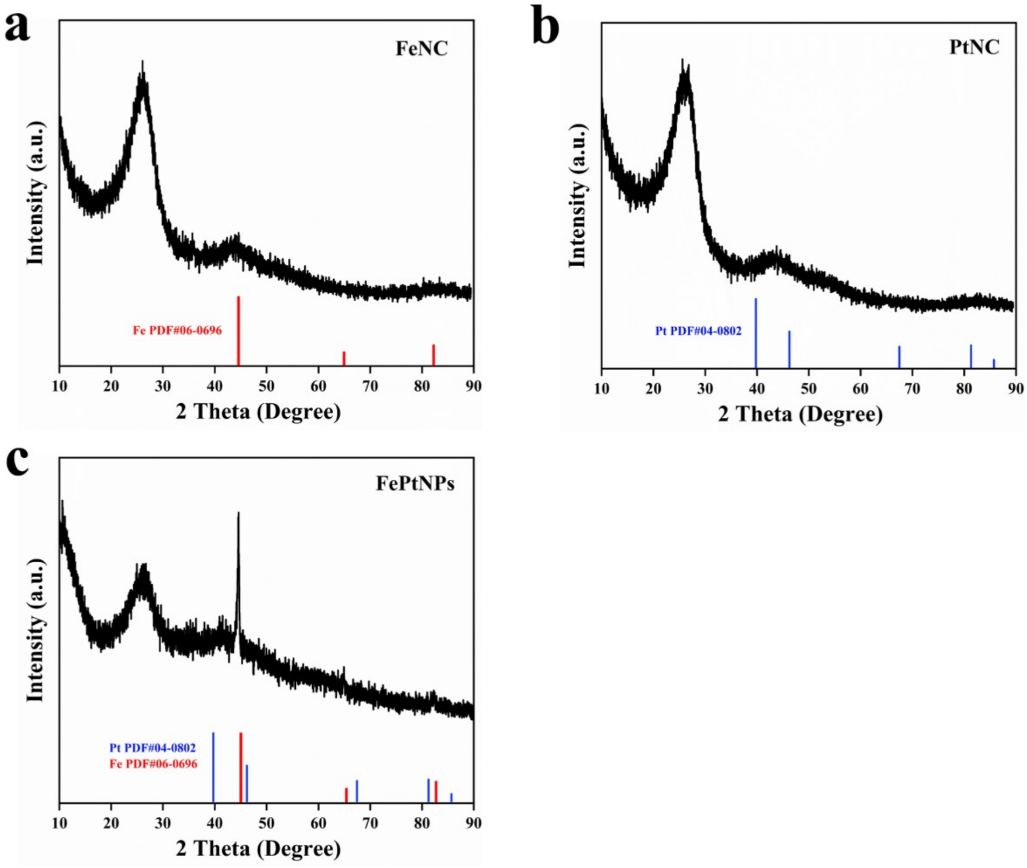

Figure S6. XRD patterns of FeNC (a), PtNC (b) and FePtNPs (c).

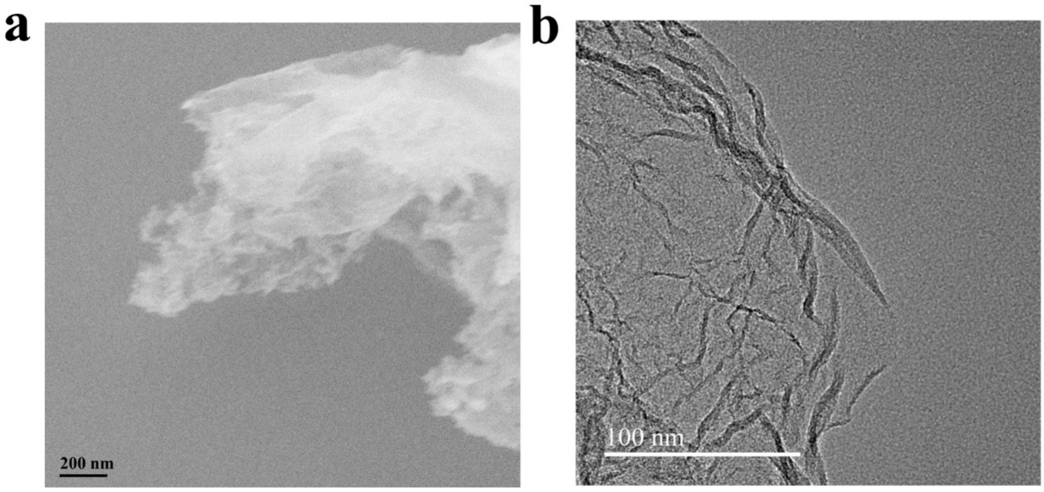

Figure S7. SEM (a) and TEM (b) images of FeNC.

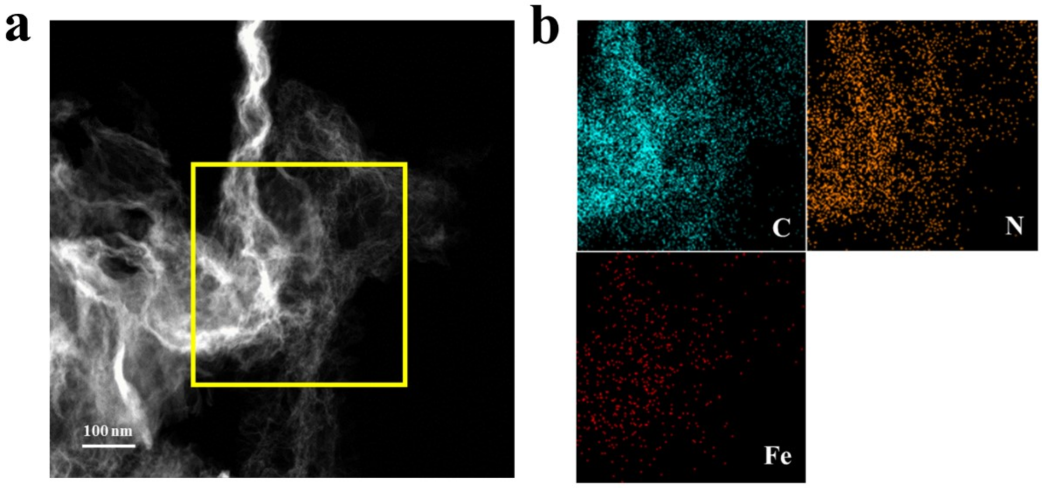

Figure S8. HAADF-STEM (a) and EDS (b) mapping images of FeNC.

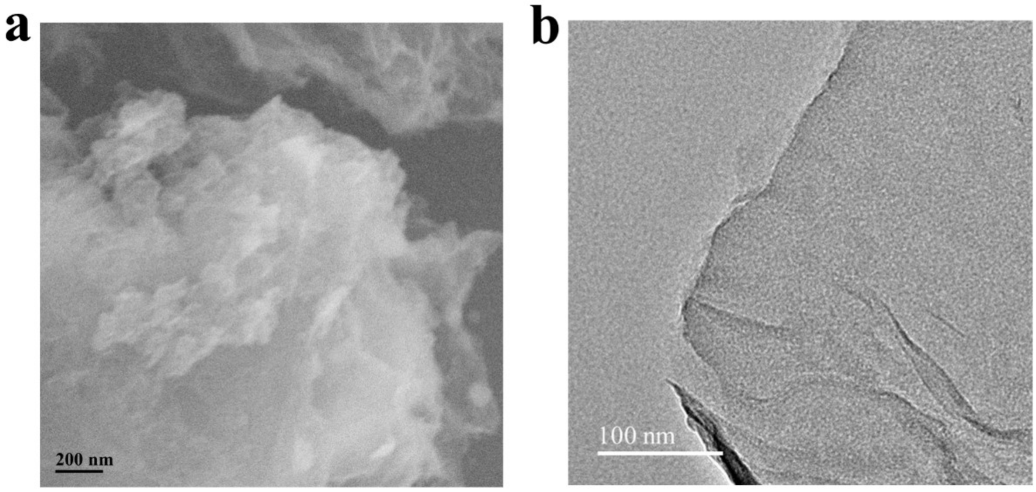

Figure S9. SEM (a) and TEM (b) images of PtNC.

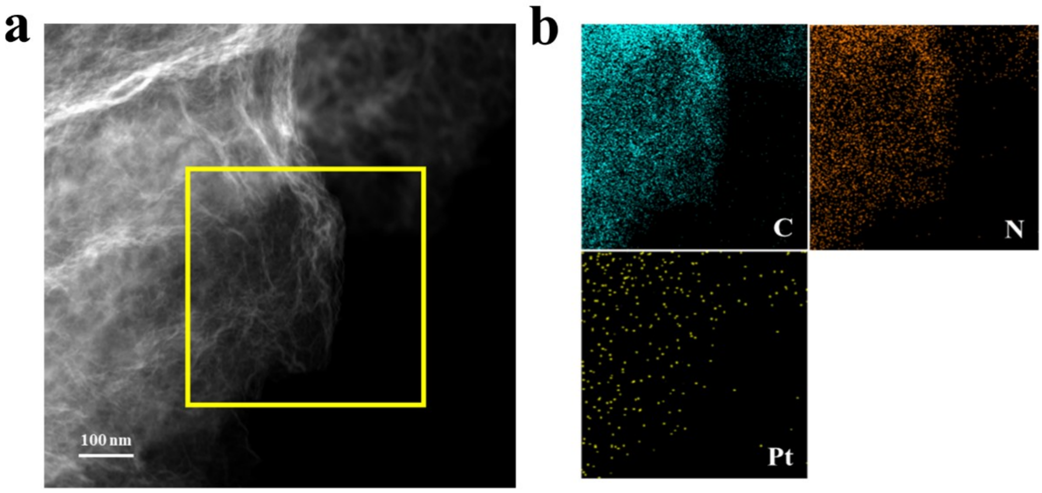

Figure S10. HAADF-STEM (a) and EDS (b) mapping images of PtNC.

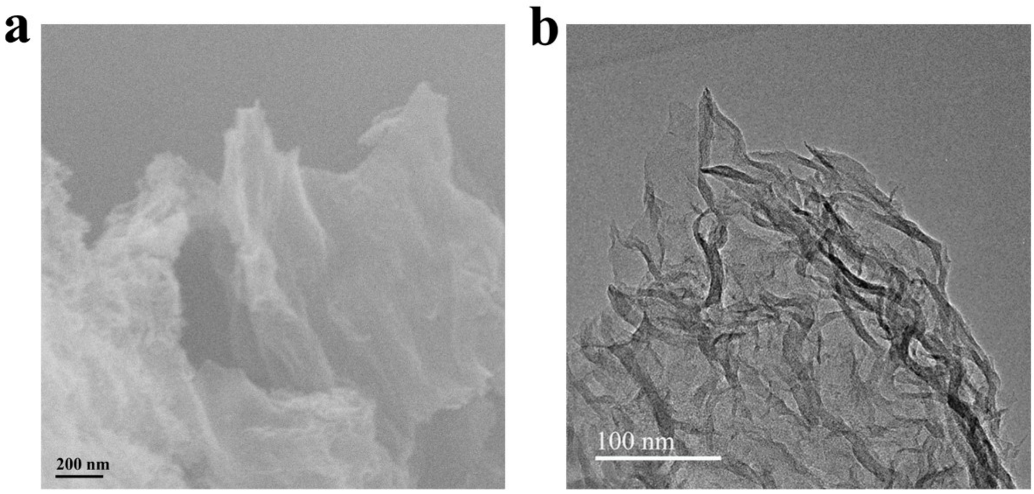

Figure S11. SEM (a) and TEM (b) images of NC.

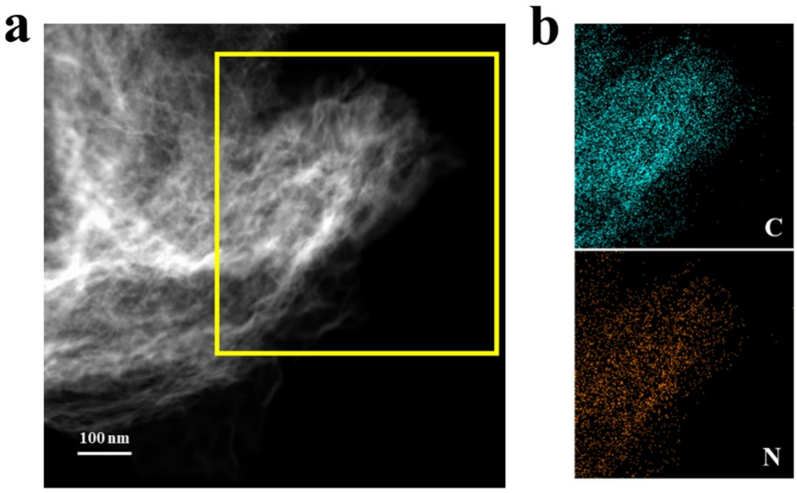

Figure S12. HAADF-STEM (a) and EDS (b) mapping images of NC.

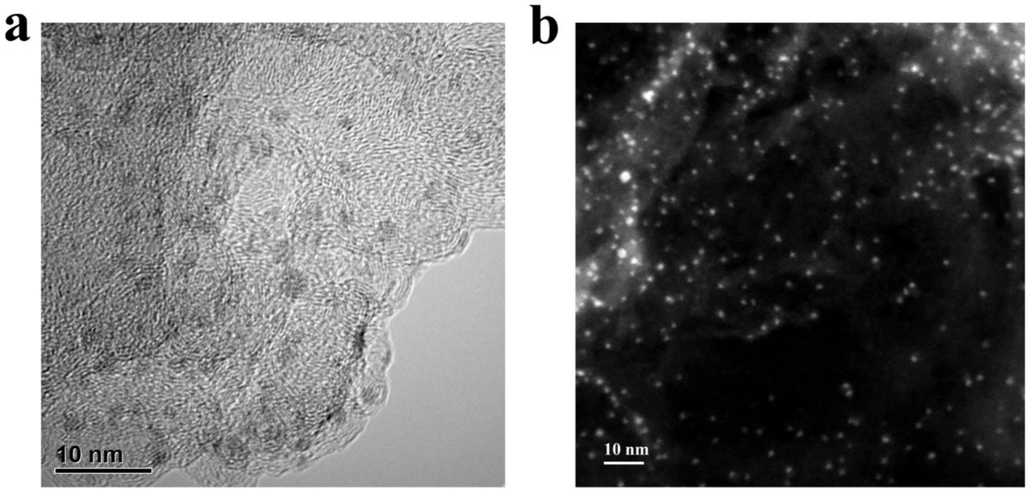

Figure S13. TEM (a) and HAADF-STEM (b) images of FePtNPs.

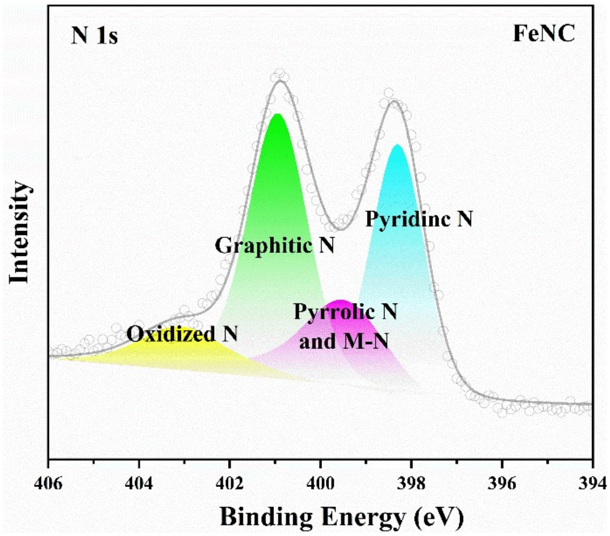

Figure S14. XPS spectra of FeNC (High-resolution N 1s spectrum).

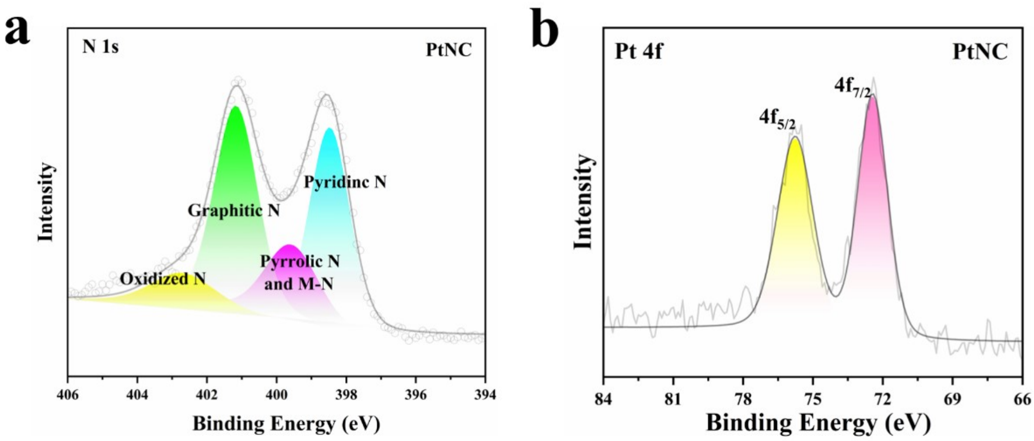

Figure S15. XPS spectra of PtNC. (a) High-resolution N 1s spectrum; (b) High-resolution Pt 4f spectrum.

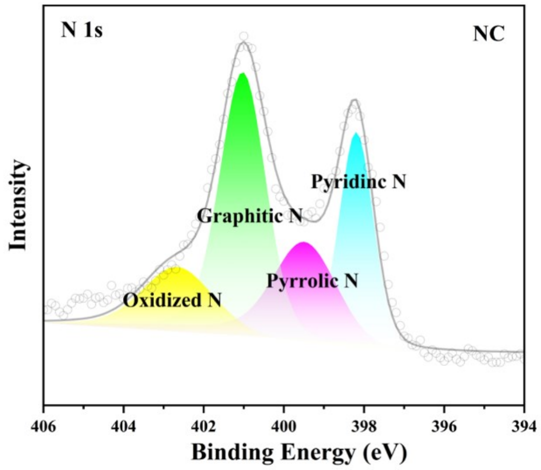

Figure S16. XPS spectrum of NC (high-resolution N 1s spectrum).

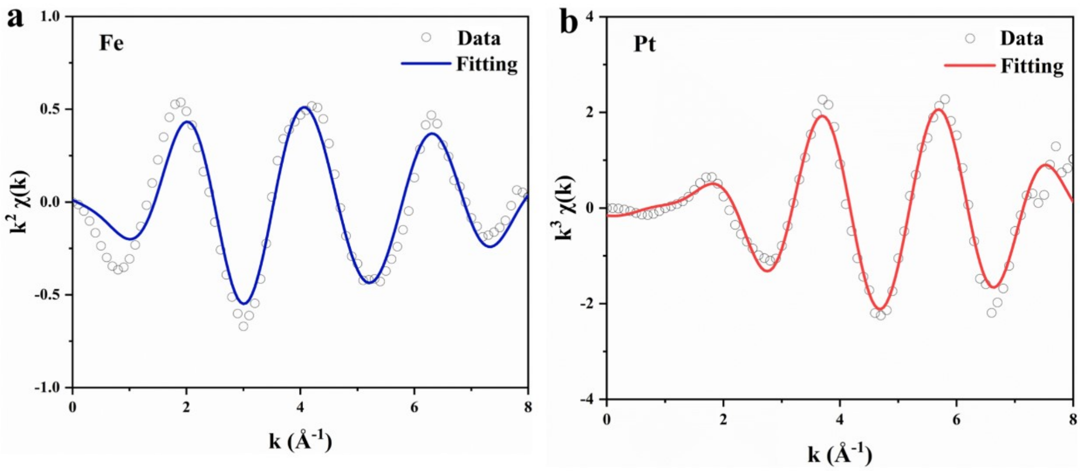

Figure S17. k space curves and corresponding fitting curves of Fe K edge (a) and Pt L_3 edge (b) of FePtNC.

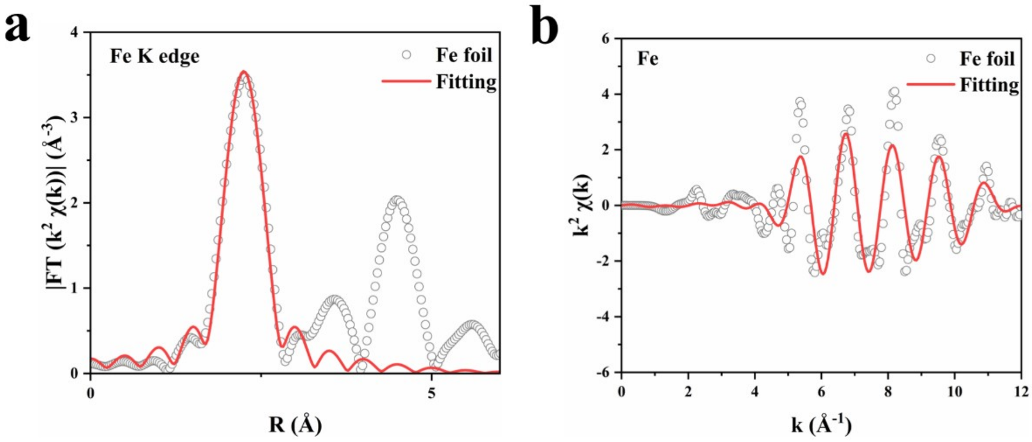

Figure S18. (a) Fourier-transform EXAFS curves and (b) k space curves and corresponding fitting curves of Fe foil.

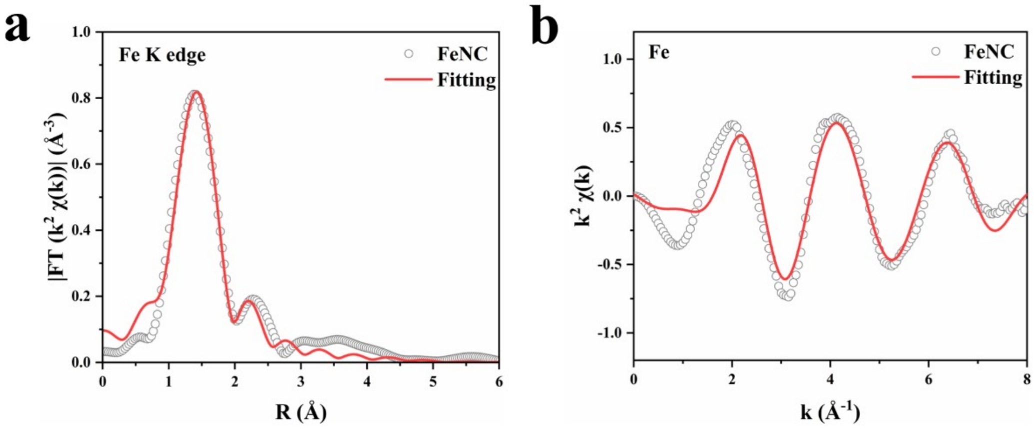

Figure S19. (a) Fourier-transform EXAFS curves and (b) k space curves and corresponding fitting curves of FeNC.

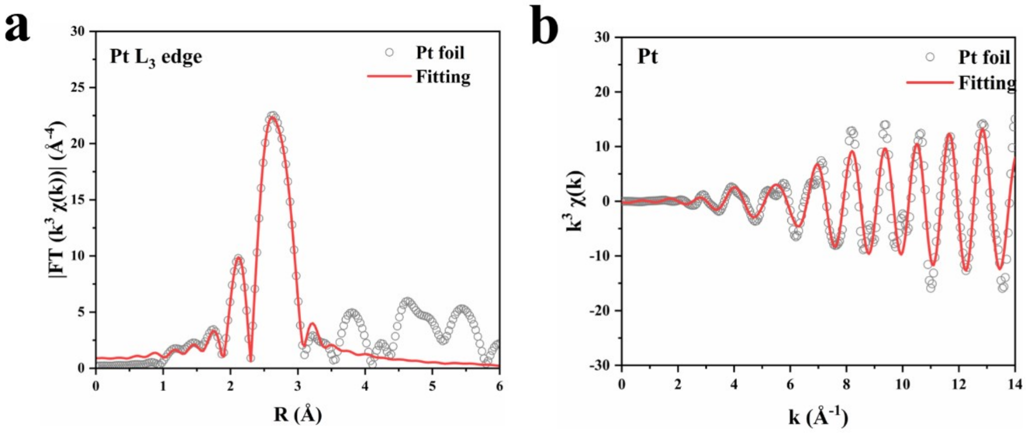

Figure S20. (a) Fourier-transform EXAFS curves and (b) k space curves and corresponding fitting curves of Pt foil.

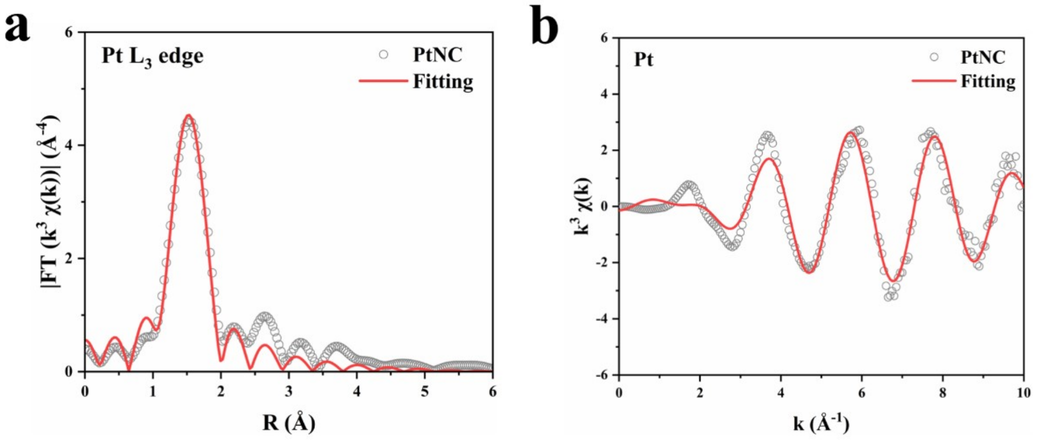

Figure S21. (a) Fourier-transform EXAFS curves and (b) k space curves and corresponding fitting curves of PtNC.

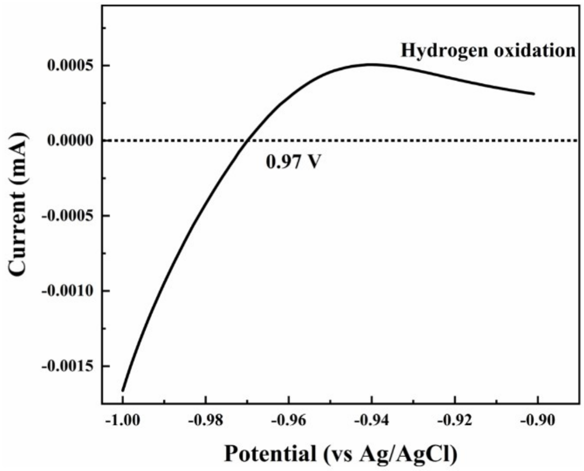

Figure S22. The potential of the reference electrode is to be converted to the reversible hydrogen electrode (RHE) potential by the following formula, $E_{(RHE)} = E_{(Ag/AgCl)} + 0.97$ V, calibrated by hydrogen oxidation reaction. LSV measurements were performed by using two platinum sheets as working electrodes in a hydrogen-saturated 0.1 M KOH solution (scan rate: 5 mV/s).

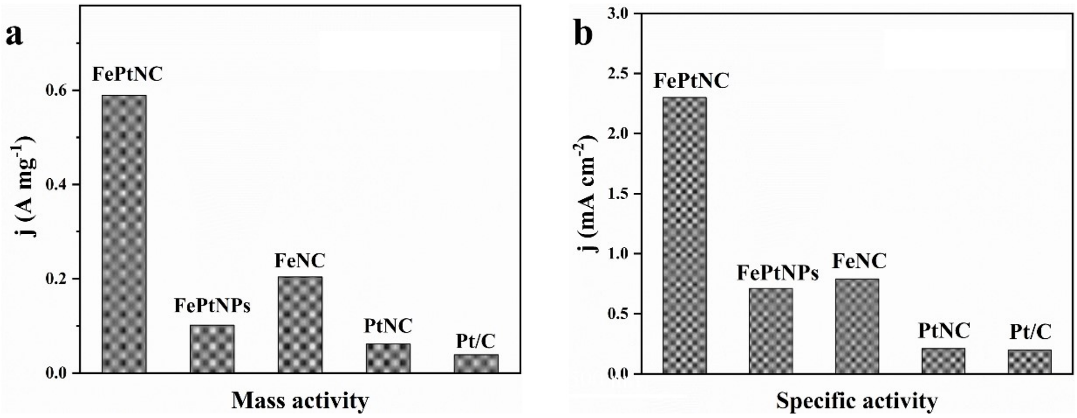

Figure S23. Column diagrams of mass activity and specific activity at 0.8 V vs. RHE.

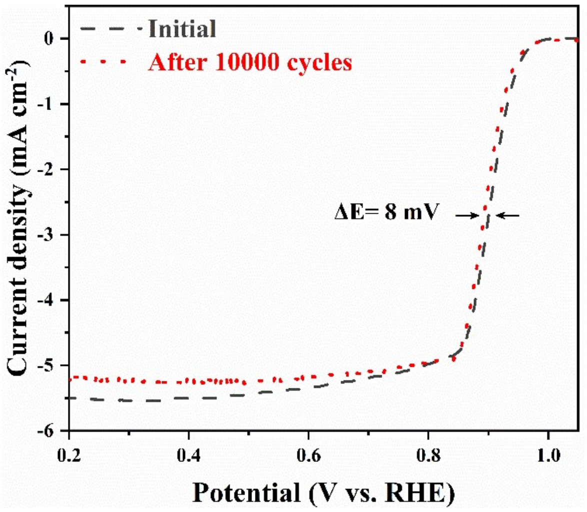

Figure S24. ORR polarization curves of FePtNC before (black line) and after cyclability (red line).

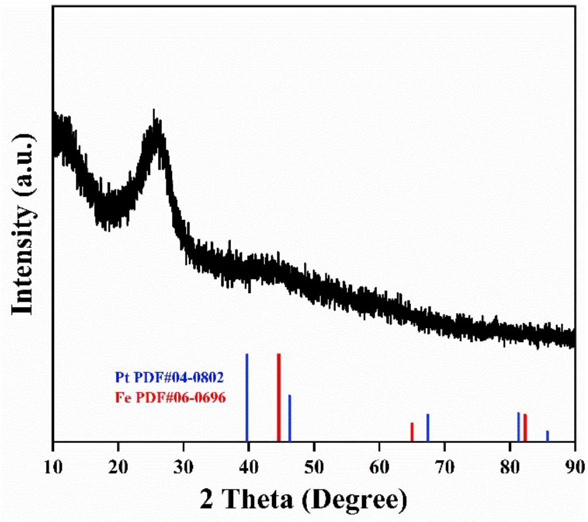

Figure S25. The XRD image of FePtNC after durability testing.

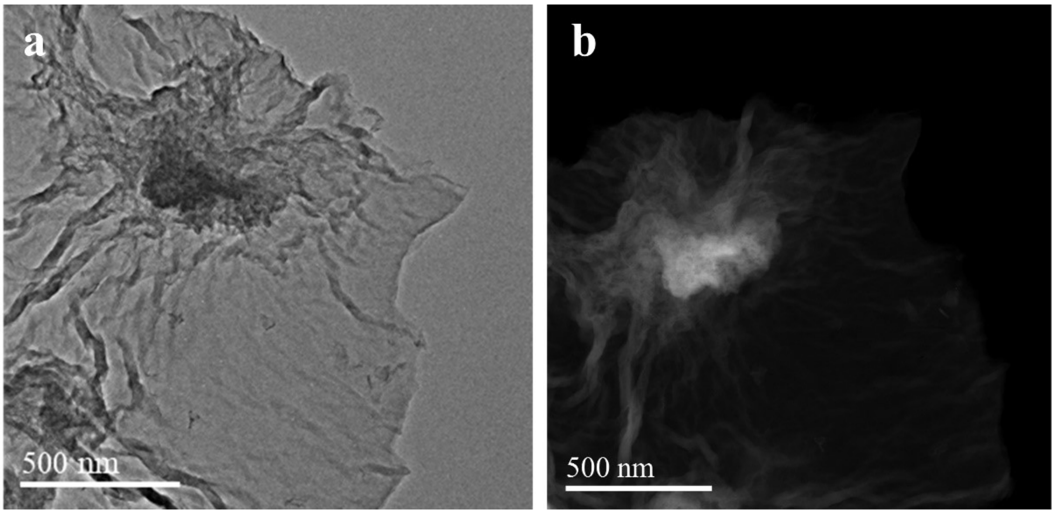

Figure S26. The TEM and HAADF STEM images of FePtNC after durability testing.

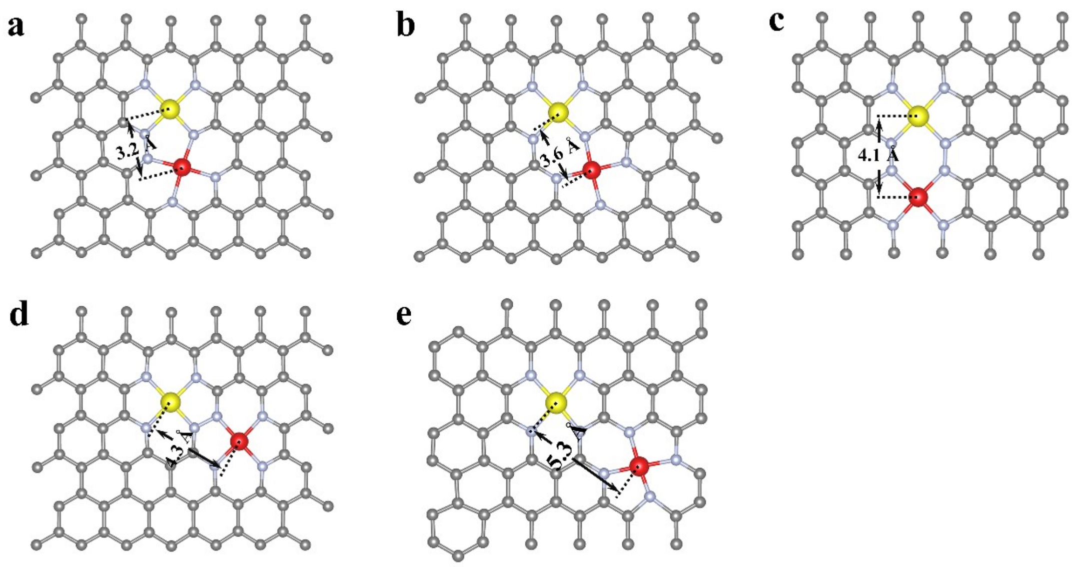

Figure S27. DFT-optimized structural models of FePtNC.

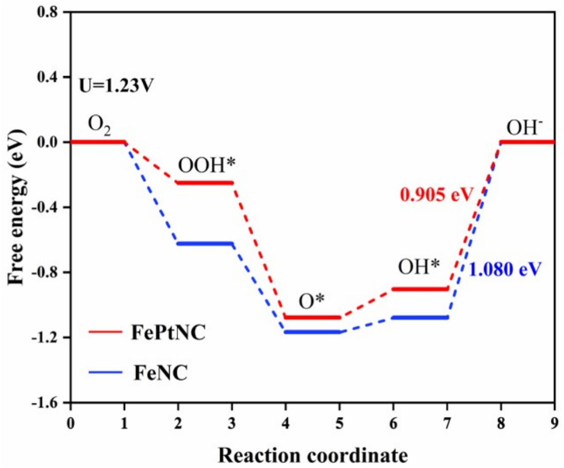

Figure S28. The DFT calculated free energy diagram of ORR for the pure Fe-N_4-Pt-N_4 model.

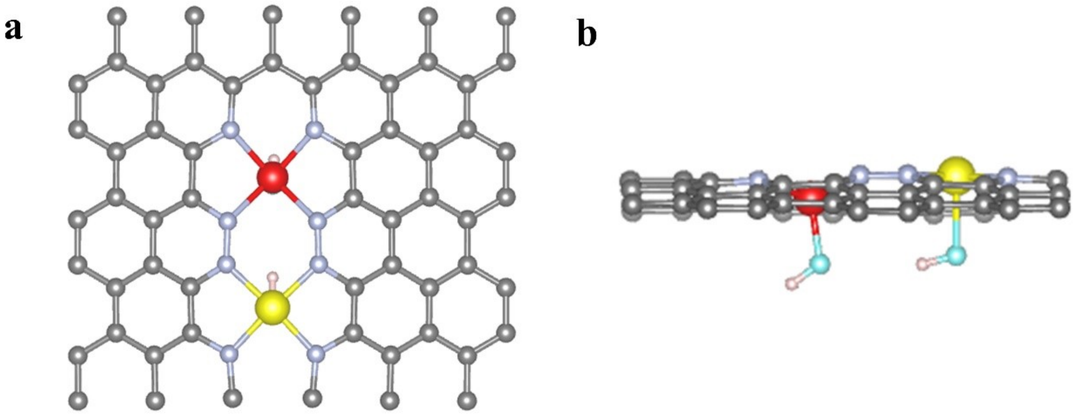

Figure S29. OH adsorption models of Fe-N_4-Pt-N_4.

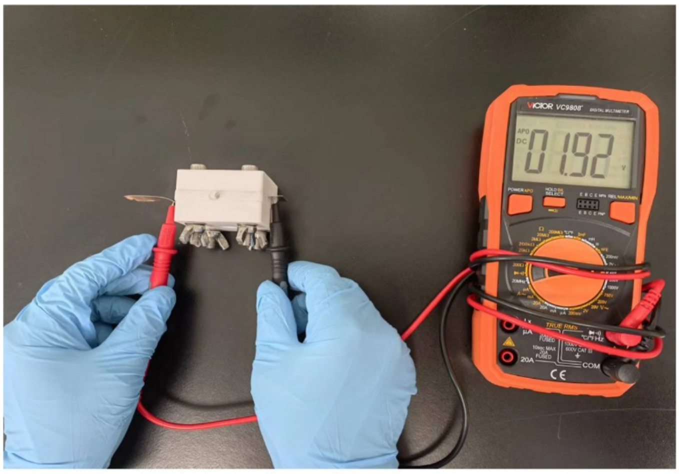

Figure S30. Photograph of commercial Pt/C based Al-air battery, with an open circuit potential voltage of 1.92 V.

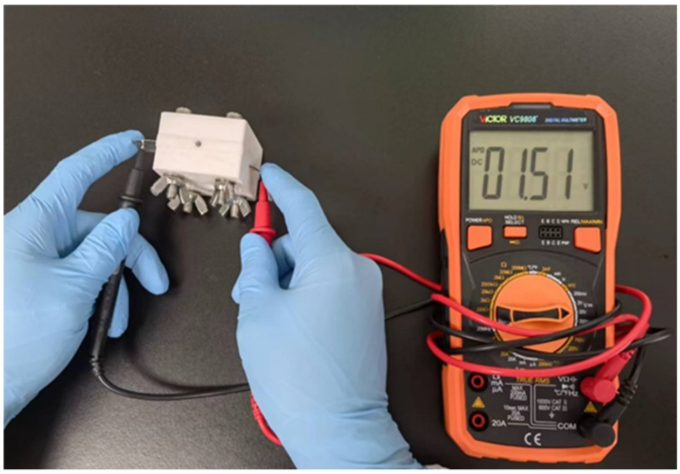

Figure S31. Photograph of commercial Pt/C based Zn-air battery, with an open circuit potential voltage of 1.51 V.

Table S1. The content of metal in the samples was obtained by the ICP-OES measurement.

Sample	Fe (wt%)	Pt(wt%)
FePtNC	2.1	1.4
FeNC	2.1	-
PtNC	-	1.9
FePtNPs	2.3	1.5
NC	-	-

Table S2. The fitting parameters and results of the EXAFS spectra of the samples at Fe K edge and Pt L_3 edge.

Sample	Edge	Path	R(Å)	CN	σ^2 *10^{-3} (Å^2)
Fe foil	Fe K edge	Fe-Fe	2.52 ± 0.01	8*	3.7
FeNC	Fe K edge	Fe-N	1.99±0.01	4.54±0.76	8.5
FePtNC	Fe K edge	Fe-N	2.01±0.02	4.54±0.76	8.4
Pt foil	Pt L_3 edge	Pt-Pt	2.76±0.02	12*	4.7
PtNC	Pt L_3 edge	Pt-N	1.96±0.02	3.72±0.71	3.3
FePtNC	Pt L_3 edge	Pt-N	2.02±0.01	3.67±0.63	6.6

R is the distance between the central atom and surrounding coordination atoms; CN is the coordination number; * indicates that this item is the known coordination number; σ^2 is the Debye-Waller factor (described the attenuation due to thermal motion).

Table S3. Comparison of ORR activity between our work and other previously published work under 0.1M KOH solution.

Catalysts	$E_{1/2}$ (vs. RHE)	Reference
FePtNC	0.90 V	This work
PtFeNC	0.895 V	[4]
FeN_4/PtN_4@NC	0.93 V	[5]
Pt_1@Fe-N-C	0.87 V	[6]
SA-PtCoF	0.88 V	[7]
Pt_1-N/BP	0.87 V	[8]
Pt_1/NPC	0.88 V	[9]
FeCo-N-HCN	0.86 V	[10]
Fe_1Se_1-NC	0.88 V	[11]
Fe–NiNC-50	0.85 V	[12]
FeNi SAs/NC	0.84 V	[13]
FeNi/N-LCN	0.85 V	[14]
FeCo−NC	0.877 V	[15]
Fe-N_4 SAs/NPC	0.885 V	[16]
Fe_1-HNC-500-850	0.85 V	[17]
S,N-Fe/N/CNT	0.85 V	[18]
Fe-N-C/MXene	0.84 V	[19]
Fe SAC/N-C	0.89 V	[20]
Fe-SAs/NSC	0.87 V	[21]
Fe/SNCFs-NH_3	0.89 V	[22]
FeN_4-Ten	0.867 V	[23]

Table S4. Comparison of Zn-air battery performance between our work and other previously published work.

Catalysts	OCP (V)	Special capacity (mAh g^{-1})	Peak power density (mW cm^{-2})	Reference
FePtNC	1.57	713	191.8	This work
PtFeNC	1.492	807	148	[4]
Fe-N_4/Pt-N_4@NC	1.48	749.8	200	[5]
SA-PtCoF	1.31	808	125	[7]
FeCo-N_x-CN	1.405	-	150	[24]
FeCo@MNC	1.41	-	115	[25]
FeNiCo@NC-P	1.36	807	112	[26]
PdMo/C	1.483	798	154.2	[27]
FeNC-S-Fe_xC/Fe	1.41	663	149.4	[28]
Fe-N_x-C	1.49	641	96.4	[29]
Fe-SAs/NPSHC	1.45	-	195	[29]
Fe SAs/N-C	1.48	636	255	[30]

References

1 B. Ravel and M. Newville, *J. Synchrotron. Radiat.*, 2005, **12**, 537-541.

2 M. Bajdich, M. García-Mota, A. Vojvodic, J. K. Nørskov and A. T. Bell, *J. Am. Chem. Soc.*, 2013, **135**, 13521-13530.

3 L. Li, J. M. P. Martirez and E. A. Carter, *ACS Catal.*, 2020, **10**, 12841-12857.

4 X. W. Zhong, S. L. Ye, J. Tang, Y. M. Zhu, D. J. Wu, M. Gu, H. Pan and B. M. Xu, *Appl. Catal., B,* 2021, **286**, 119891.

5 A. Han, X. Wang, K. Tang, Z. Zhang, C. Ye, K. Kong, H. Hu, L. Zheng, P. Jiang, C. Zhao, Q. Zhang, D. Wang and Y. Li, *Angew. Chem., Int. Ed.,* 2021, **60**, 19262-19271.

6 X. Zeng, J. Shui, X. Liu, Q. Liu, Y. Li, J. Shang, L. Zheng and R. Yu, *Adv. Energy Mater.*, 2018, **8**, 1701345.

7 Z. Li, W. Niu, Z. Yang, N. Zaman, W. Samarakoon, M. Wang, A. Kara, M. Lucero, M. V. Vyas, H. Cao, H. Zhou, G. E. Sterbinsky, Z. Feng, Y. Du and Y. Yang, *Energy Environ. Sci.*, 2020, **13**, 884-895.

8 J. Liu, M. Jiao, L. Lu, H. M. Barkholtz, Y. Li, Y. Wang, L. Jiang, Z. Wu, D.-j. Liu, L. Zhuang, C. Ma, J. Zeng, B. Zhang, D. Su, P. Song, W. Xing, W. Xu, Y. Wang, Z. Jiang and G. Sun, *Nat. Commun.*, 2017, **8**, 15938.

9 T. Li, J. Liu, Y. Song and F. Wang, *ACS Catal.*, 2018, **8**, 8450-8458.

10 H. Li, Y. Wen, M. Jiang, Y. Yao, H. Zhou, Z. Huang, J. Li, S. Jiao, Y. Kuang and S. Luo, *Adv. Funct. Mater.*, 2021, **31**, 2011289.

11 Z. Chen, X. Su, J. Ding, N. Yang, W. Zuo, Q. He, Z. Wei, Q. Zhang, J. Huang and Y. Zhai, *Appl. Catal., B,* 2022, **308**, 121206.

12 X. Zhu, D. Zhang, C.-J. Chen, Q. Zhang, R.-S. Liu, Z. Xia, L. Dai, R. Amal and X. Lu, *Nano Energy*, 2020, **71**, 104597.

13 D. Yu, Y. Ma, F. Hu, C.-C. Lin, L. Li, H.-Y. Chen, X. Han and S. Peng, *Adv. Energy Mater.*, 2021, **11**, 2101242.

14 X. Li, Y. Liu, H. Chen, M. Yang, D. Yang, H. Li and Z. Lin, *Nano Lett.*, 2021, **21**, 3098-3105.

15 Y. He, X. Yang, Y. Li, L. Liu, S. Guo, C. Shu, F. Liu, Y. Liu, Q. Tan and G. Wu, *ACS Catal.*, 2022, **12**, 1216-1227.

16 Y. Pan, S. Liu, K. Sun, X. Chen, B. Wang, K. Wu, X. Cao, W.-C. Cheong, R. Shen, A. Han, Z. Chen, L. Zheng, J. Luo, Y. Lin, Y. Liu, D. Wang, Q. Peng, Q. Zhang, C. Chen and Y. Li, *Angew. Chem., Int. Ed.*, 2018, **57**, 8614-8618.

17 X. Zhang, S. Zhang, Y. Yang, L. Wang, Z. Mu, H. Zhu, X. Zhu, H. Xing, H. Xia, B. Huang, J. Li, S. Guo and E. Wang, *Adv. Mater.*, 2020, **32**, 1906905.

18 P. Chen, T. Zhou, L. Xing, K. Xu, Y. Tong, H. Xie, L. Zhang, W. Yan, W. Chu, C. Wu and Y. Xie, *Angew. Chem., Int. Ed.*, 2017, **56**, 610-614.

19 L. Jiang, J. Duan, J. Zhu, S. Chen and M. Antonietti, *Acs Nano.*, 2020, **14**, 2436-2444.

20 Y. Lin, P. Liu, E. Velasco, G. Yao, Z. Tian, L. Zhang and L. Chen, *Adv. Mater.*, 2019, **31**, 1808193.

21 J. Zhang, Y. Zhao, C. Chen, Y.-C. Huang, C.-L. Dong, C.-J. Chen, R.-S. Liu, C. Wang, K. Yan, Y. Li and G. Wang, *J. Am. Chem. Soc.*, 2019, **141**, 20118-20126.

22 L. Yang, X. Zhang, L. Yu, J. Hou, Z. Zhou and R. Lv, *Adv. Mater.*, 2022, **34**, 2105410.

23 B. Ji, J. Gou, Y. Zheng, X. Zhou, P. Kidkhunthod, Y. Wang, Q. Tang and Y. Tang, *Adv. Mater.*, 2022, **34**, 2202714.

24 S. Li, C. Cheng, X. Zhao, J. Schmidt and A. Thomas, *Angew. Chem., Int. Ed.*, 2018, **57**, 1856-1862.

25 C. Li, M. Wu and R. Liu, *Appl. Catal., B*, 2019, **244**, 150-158.

26 D. Ren, J. Ying, M. Xiao, Y.-P. Deng, J. Ou, J. Zhu, G. Liu, Y. Pei, S. Li, A. M. Jauhar, H. Jin, S. Wang, D. Su, A. Yu and Z. Chen, *Adv. Funct. Mater.*, 2020, **30**, 1908167.

27 M. Luo, Z. Zhao, Y. Zhang, Y. Sun, Y. Xing, F. Lv, Y. Yang, X. Zhang, S. Hwang, Y. Qin, J.-Y. Ma, F. Lin, D. Su, G. Lu and S. Guo, *Nature*, 2019, **574**, 81-85.

28 Y. Qiao, P. Yuan, Y. Hu, J. Zhang, S. Mu, J. Zhou, H. Li, H. Xia, J. He and Q. Xu, *Adv. Mater.*, 2018, **30**, 1804504.

29 Y. Chen, S. Ji, S. Zhao, W. Chen, J. Dong, W.-C. Cheong, R. Shen, X. Wen, L. Zheng, A. I. Rykov, S. Cai, H. Tang, Z. Zhuang, C. Chen, Q. Peng, D. Wang and Y. Li, *Nat. Commun.*, 2018, **9**, 5422.

30 Z. Yang, Y. Wang, M. Zhu, Z. Li, W. Chen, W. Wei, T. Yuan, Y. Qu, Q. Xu, C. Zhao, X. Wang, P. Li, Y. Li, Y. Wu and Y. Li, *ACS Catal.*, 2019, **9**, 2158-2163.
